# Policy insights for national school meals programmes: Annual Research Statements to the members of the School Meals Coalition - 2022, 2023, and 2024

**DOI:** 10.3389/fpubh.2025.1416165

**Published:** 2025-07-18

**Authors:** Research Consortium for School Health and Nutrition, Donald A.P. Bundy, Linda Schultz, Kate Morris, Jasmine Catmull, Georgia Crowley, Sarah Vickers, Patricia Eustachio Colombo, Muna Osman, Gilbert Miki, Edward Makumbe, Noam Angrist, Stéphane Verguet, Sylvie Avallone, Heli Kuusipalo, Aurélie Fernandez, R. Gopinath, Abimbola Adesanmi, Samrat Singh, Jean D’Amour Manirere, Silvia Pastorino, Jacqueline Kungú, Dirce Maria Lobo Marchioni, Semiramis Domene

**Affiliations:** Department of Population Health, Research Consortium for School Health and Nutrition, London School of Hygiene and Tropical Medicine, London, United Kingdom

**Keywords:** school health and nutrition, school meals, School Meals Coalition, human capital, school-age children, adolescents, education policy

## Abstract

The scale and near universality of school closures in response to the COVID-19 pandemic highlighted the vital role schools play in protecting the health and wellbeing of learners. This experience strengthened the resolve of countries to re-establish and increase their investment in the education and wellbeing of children as part of building back from the pandemic, resulting in the creation of a global School Meals Coalition (SMC) to help achieve that ambition by 2030. The Research Consortium for School Health and Nutrition was launched in 2021 to provide independent, evidence-based guidance on effective policymaking on school health and nutrition programming to the (now) 109 member states of the Coalition. Guided by a 10-year independent research strategy, the Research Consortium operates as a global network of academics and scholars and consolidates research findings into policy insights that offer actionable approaches to strengthen the quality, efficiency, and coverage of national programmes. With input from over 1,200 Global Academy members from more than 110 countries, the Research Consortium develops an Annual Research Statement on the policy implications of the emerging research in this area, which is presented to policymakers annually at a global convening of the SMC member states. This paper introduces the Research Consortium’s Annual Research Statements for years 2022, 2023, and 2024, which report new programmatic and policy insights as well as accumulating and evolving understanding of the research on school health and nutrition.

## Introduction

The Research Consortium for School Health and Nutrition is the first initiative of the School Meals Coalition, created at the request of the (now) 109 member states to assemble and share policy-relevant evidence on school meals to support priority setting and decision making in this area. This paper introduces the Research Consortium’s Annual Research Statements for years 2022, 2023, and 2024, which report new programmatic and policy insights as well as accumulating and evolving understanding of the research on school health and nutrition. The latest statement was presented at the School Meals Coalition (SMC) Ministerial Summit hosted by Kenya in October 2024, and builds on the available research since the 2023 SMC Global Summit hosted by France and the 2022 SMC Ministerial Meeting hosted by Finland. In an effort to support policy makers with well-informed priority setting, the 2024 Statement concludes by suggesting five priority actions to increase the reach, quality, and comprehensiveness of national school meals programmes.

## The creation of a Coalition with the specific aim of rebuilding the school-based health and nutrition services in response to the global pandemic

The closure of schools worldwide amidst the COVID-19 pandemic precipitated the largest education crisis in history, leaving 1.6 billion children out of school. The pandemic was first recognized in January 2020, and by April 2020, schools had closed in 199 countries and students were required to remain at home to minimize the transmission of coronavirus. These closures immediately excluded 1.5 billion children not only from access to education but also from the health and well-being services routinely provided through schools.

While some children in high- and middle-income countries continued their education through distance learning tools, a majority did not. In the Africa region, for example, less than 10 percent of children had access to digital sources for distance education (1). In some countries, schools re-opened relatively quickly, yet for many there was a disruptive cycle of re-opening and re-closing, and for some, this withdrawal of schooling was long-term: for example, schools in Uganda and the Philippines only reopened more than two years later. In high-income countries, it is now recognized that generations of children have lost out on educational opportunities that will have life-long consequences, while in low-income countries the World Bank estimates that the already very poor level of educational achievement has deteriorated further: the proportion of children in Africa able to read a simple age-appropriate sentence declined from 70 percent to 53 percent, as compared to before the pandemic (2).

From the perspective of the wellbeing of children, at the height of the pandemic, an estimated 370 million children in 161 countries lost access to what was for many their only dependable meal of the day (3). The global pandemic also had negative sexual and reproductive health consequences, as well as negative schooling outcomes. In Kenya, for example, girls had twice the risk of falling pregnant prior to completing secondary school and three times the risk of school dropout (4). A Pulse survey by WHO in early 2021 found that, after mental health, neglected tropical diseases (NTDs) were the most frequently affected public health service, as mass drug administration campaigns targeting schoolchildren are typically delivered in school settings (5).

**Figure 1:**
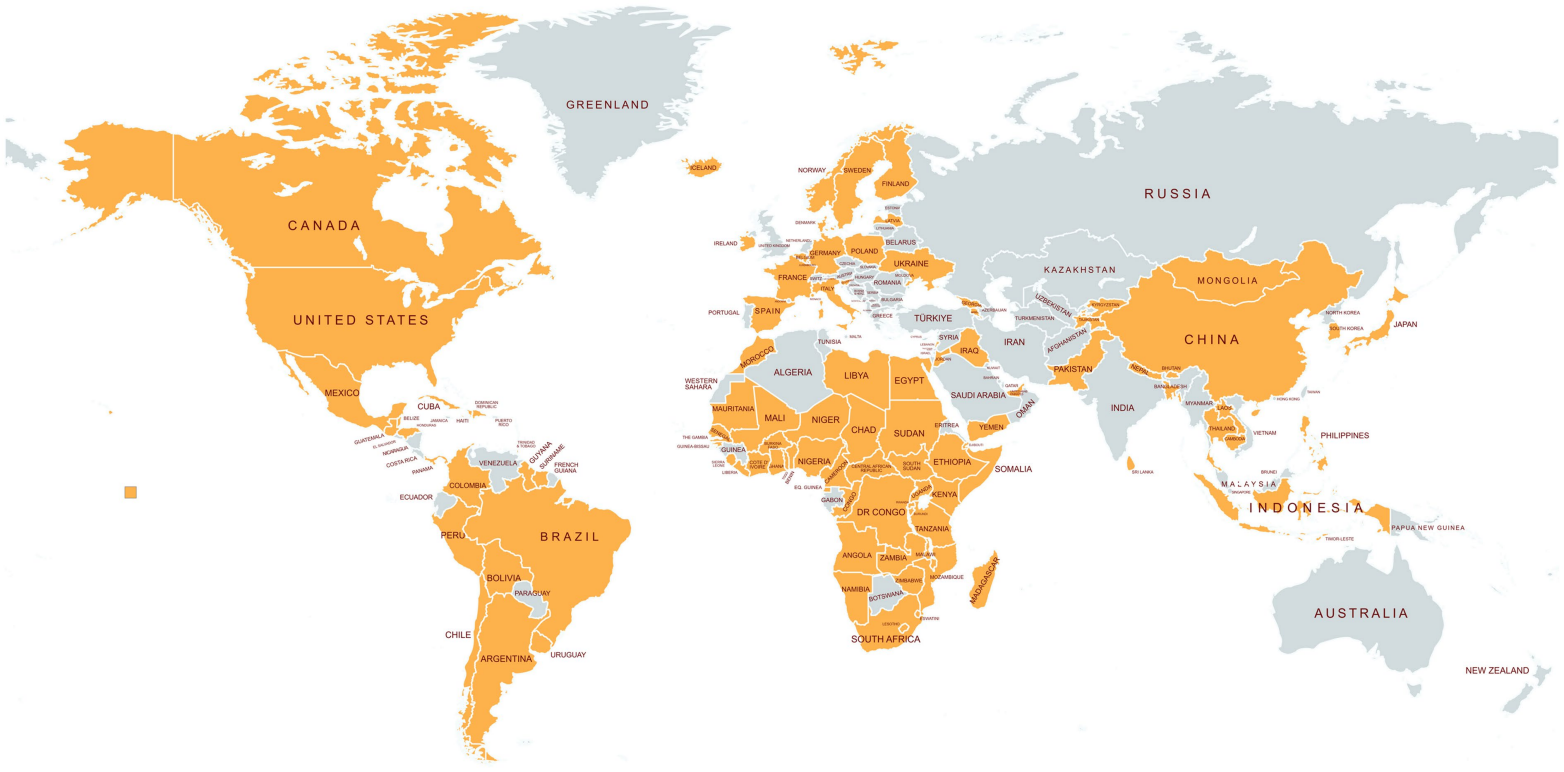
109 countries have signed the declaration of commitment to the School Meals Coalition, as of July 2025. Map created using Mapchart.net.

The experience of having service delivery interrupted by prolonged school closures, and the inadequacy of most health systems in serving school-age children and adolescents, led to national political leaders at the highest levels of government forming a global coalition at the 2021 UN Food Systems Summit. This coalition was established with the specific aims of rebuilding the school-based services that had been severely damaged by the pandemic closures and ensuring the wellbeing of current and future generations of school children. Today, 109 countries, comprising 67 percent of the world’s population (Figure 1), have created the School Meals Coalition with three specific goals: (1) to restore national school meals and complementary school health programmes to pre-pandemic coverage by 2023; and, by 2030, (2) to develop new approaches to reach an additional 73 million of the most in-need children who had not previously been reached; and (3) to raise the quality of school health and nutrition programmes globally (6). This vision is supported by six regional bodies^1^ and 145 organizations around the world, including multilateral banks such as the Inter-American Development Bank, Islamic Development Bank, and the World Bank. At the UN Food Systems Stocktaking Moment +2 in 2023, the School Meals Coalition was recognized as the most substantial coalition to arise from the Food Systems Summit.

Governments and international development organizations are delivering on this response. Despite constraints on public finances due to the health and economic costs of the COVID-19 pandemic and ongoing global conflict, low-income countries have increased the proportion of the costs of school meals from domestic budgets: up to 45 percent from 30 percent pre-pandemic. Benin, for example, has announced a national budget commitment of USD 270 million to scale up the national programme from 75 percent to 100 percent coverage by 2026. Rwanda has reached universal coverage of school meals, increasing its support for 640,000 children in 2020 to 3.8 million in 2022. High-income countries are similarly committing to more resilient school meals programmes. France introduced a scheme to subsidize lunchtime meals for the most vulnerable students, with meals costing a maximum of €1, and the United States unveiled a new National Strategy on Hunger, Nutrition and Health, which includes the objective of expanding free school meals to all children (7).

## The creation of a Research Consortium to build the global evidence base on school health and nutrition

Recognizing the fundamental importance of good science to help countries achieve these goals, the Coalition created a Research Consortium for School Health and Nutrition to provide independent scientific evidence on which programmatic actions to prioritize, based on their impact and value-for-money. The Research Consortium was launched in May 2021 with the endorsement of the heads of five UN agencies concerned with the wellbeing of children: WHO, WFP, FAO, UNESCO, and UNICEF (8).

With a small Secretariat based at the London School of Hygiene & Tropical Medicine, the Research Consortium operates as a global network of academics and scholars to ensure policymakers, parliamentarians, and practitioners from School Meals Coalition member states have access to high-quality evidence and actionable guidance on school health and nutrition. Guided by a 10-year research strategy, the Research Consortium aims to build a global evidence base on school health and nutrition until 2030 through two approaches:

1. Providing evidence on the effectiveness of school meals programmes for learning, social, and physical outcomes of children and adolescents across the world to make the case for investment in school-based health and nutrition programmes; and
2. Providing policymakers and parliamentarians with programmatic guidance on the optimal policies to be implemented with regard to health, nutrition, and education.

**Figure 2:**
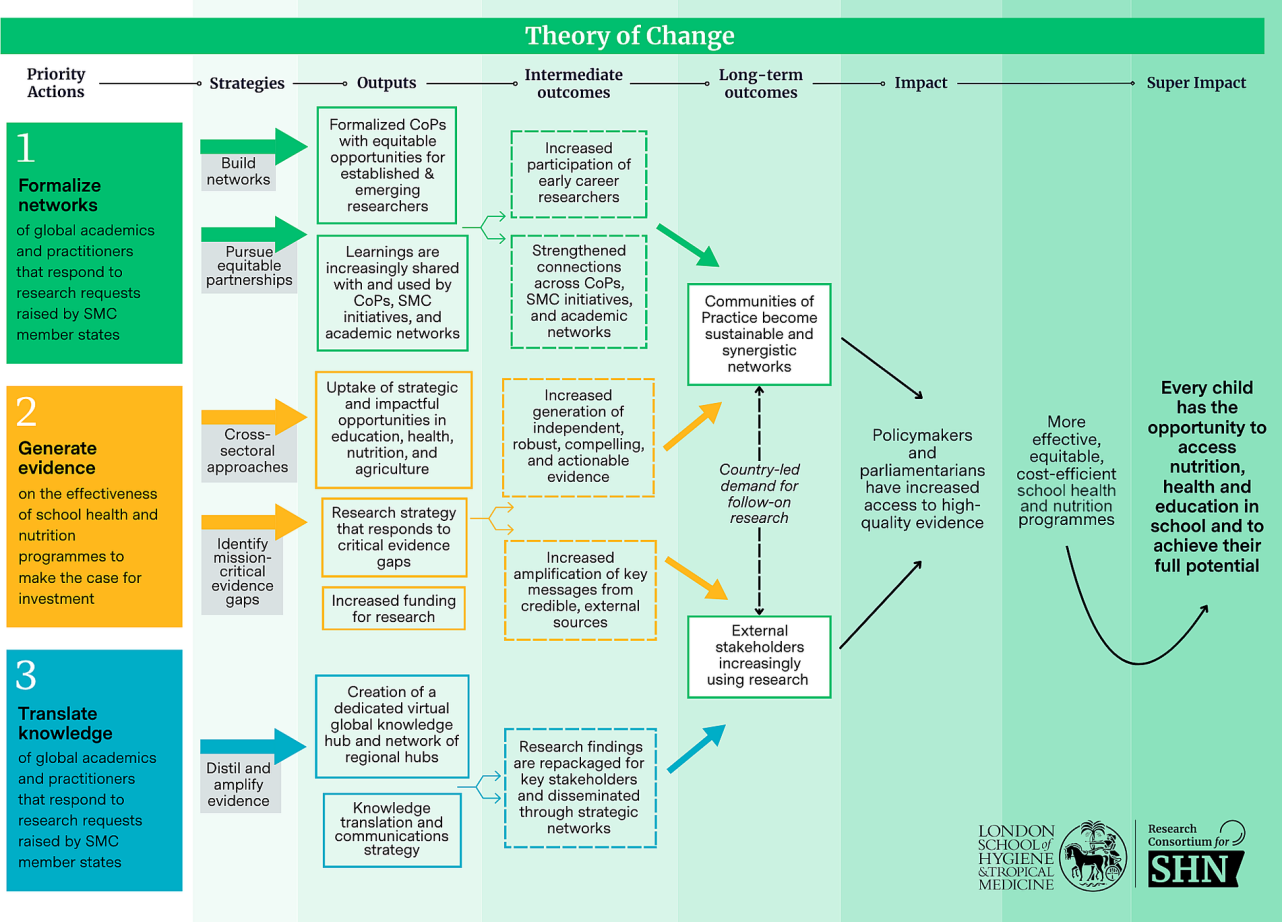
Research Consortium theory of change.

The Research Consortium’s research strategy is underpinned by a theory of change which is complementary to that developed for the School Meals Coalition, to reflect the shared objective of ensuring every child can access good nutrition, health, and education in school so that they can achieve their full potential. As shown in Figure 2, the Research Consortium’s theory of change is structured around three priority actions: formalize networks; generate evidence; and translate knowledge. These priority actions are supported by strategic approaches to help achieve the intended outputs and outcomes along the results chain.

### Priority action 1

**Formalize networks of global academics and key stakeholders that respond to research requests raised by Coalition member states.**

#### Strategic approaches

##### Identify, link and strengthen existing networks and pursue equitable partnerships

**Figure 3:**
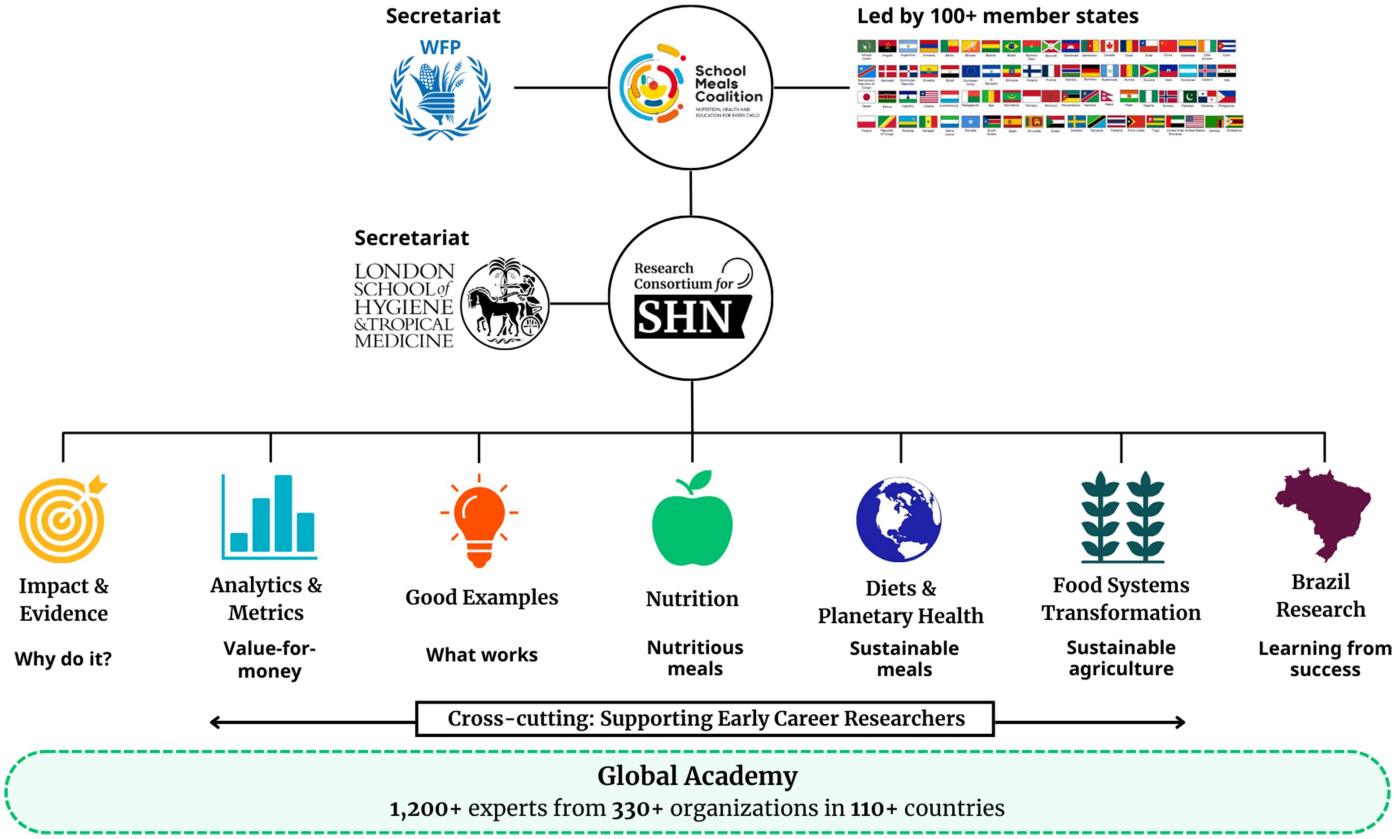
Research Consortium’s organizational structure.

In alignment with the first priority action area, the Research Consortium works through a global Community of Practice model to ensure equitable contribution to thought leadership across geographies and maintain independence of the research process and findings (Figure 3). The Communities of Practice currently lead research in thematic areas corresponding to the evidence gaps identified by member states as necessary to strengthen national policies targeting school-based programming for health and nutrition.^2^ Currently, the six operational Communities of Practices focus on the following research areas: (i) *Impact & Evidence:* assessing the impact of school-based health interventions on key development outcomes, using systematic reviews; (ii) *Analytics & Metrics:* estimating the value-for-money of school health interventions and their impact on education outcomes; (iii) *Good Examples:* documenting good practices from national school meal programmes across all the member countries of the SMC; (iv) *Nutrition:* creating and curating evidence on the nutrition of school children and adolescents, and identifying key nutrition indicators for these age-groups; (v) *Food System Transformation:* evaluating critical pathways in the production, procurement and distribution of school meals to drive sustainable food systems transformation; and (vi) *Diets & Planetary Health*: exploring the nature and impact of nutrient-dense, sustainably sourced school meals menus for improving both human and planetary health. Additional Communities of Practice are anticipated as additional thematic issues emerge. For example, a seventh Community of Practice, led by researchers in Brazil is currently under development. Supporting opportunities for early career researchers remains a cross-cutting priority for all Communities of Practice.

The Research Consortium is keenly aware of the risks when decision-making power and research efforts are overly concentrated in the Global North, and has therefore intentionally and deliberately set-up these processes so that they are global in their engagement. Underpinning the Communities of Practice is the Consortium’s “Global Academy” of more than 1,200 professors, scholars, and practitioners in school health and nutrition, now representing more than 110 countries and 330 organizations across the world. The Research Consortium also liaises with existing regional and country knowledge hubs to co-create contextually relevant evidence and guidance for SMC member states. This diverse network fosters a two-way exchange of knowledge between the Consortium and its stakeholders, where global expertise informs local practice, and insights from on-the-ground experiences shape the broader research and policy agenda.

### Priority action 2

**Generate evidence on the effectiveness of school health and nutrition programmes to make the case for investment.**

#### Strategic approaches

##### Cross-sectoral approaches and identify mission critical evidence gaps

In alignment with the second priority action area, the Research Consortium takes a global approach to guide academics, policymakers, and parliamentarians, with the research informed by country-based analyses and meta-analyses. Filling these evidence gaps involves a process of assembling all existing research in the area and conducting new research where necessary, with these efforts led by the global Communities of Practice.

To illustrate this approach, the Research Consortium’s Planet-Friendly Diets and Food Systems Transformation Communities of Practice led an analysis over a 12-month period to better understand the potential to effect change at scale through the implementation of planet-friendly school meals policies. To explore this question, the Consortium engaged 87 institutions globally and 164 experts to identify planet-friendly policies that governments can introduce to positively influence school meals and food systems across income settings (9). The analysis, which built upon original mathematical modeling analyses and more than 40 case studies, concluded that governments can make immediate policy changes to improve both health and the environment across different income settings through school meals menus, fuel-efficient preparation methods, improved waste management, and food education. These actions can collectively create demand from the agricultural sector for school foods from ecologically sustainable local farm systems. In December 2023, the Research Consortium presented its findings for formal discussion at the COP28 climate conference in Dubai, UAE. Several national governments, including Kenya, Rwanda, and Sierra Leone, announced their intention to implement the approaches described in the White Paper to improve child and planet health. The Consortium is continuing to disseminate the White Paper’s key findings through a webinar series, a forthcoming special issue in *The Lancet Planetary Health*, and at high-level global events, including at COP29 in Baku, Azerbaijan.

In the three years since its launch, the Research Consortium has published more than 55 papers across some 20 leading journals to build evidence on school health. In addition, members of the Consortium have served as Commissioners on high-impact analyses, including The *Lancet* Commission on Investing in Health (CIH 3.0) and the EAT-*Lancet* 2.0 (10); provided technical advice to the editorial teams of international publications such as WFP’s *State of School Feeding Worldwide 2022* and its forthcoming 2024 edition (11); UNESCO’s *Ready to Learn and Thrive: School health and nutrition around the world and its Education and Nutrition*: Learn to Eat GEM report (12, 45); evidence-based memos for the Global Education Commission (13); the Fourth Edition of the World Bank’s Disease Control Priorities (7, 14) and the biennial reports by the African Union on home-grown school feeding (15, 16).

A central activity of the Research Consortium is to develop an Annual Research Statement to distill the evolving and accumulating programmatic and policy insights generated by the global Communities of Practice. The Statement is presented to policymakers at the annual convenings of SMC member states, and concludes with concrete approaches to translate these insights into policy action and priority setting. Broad support from donors enables the Consortium Secretariat and Communities of Practice to collaborate with regional and country hubs in the translation of research findings for regional, national, and sub-national decisionmakers. The 2024 Research Statement is presented in its entirety in Part 2 of this paper, with the 2023 and 2022 Research Statements included as Supplementary Annexes.

### Priority action 3

**Translate knowledge in collaboration with a broad network of actors, ensuring global access.**

#### Strategic approaches

##### Distil and amplify evidence

In line with the third priority action area of the theory of change, a key responsibility of the Research Consortium is to ensure research outputs are communicated to school health and nutrition stakeholders. To date, the Consortium has hosted 36 virtual events attracting over 4,000 experts from more than 100 countries. Members of the Research Consortium have also been invited to speak at more than 60 externally-led national, regional, and international events within the school health and nutrition arena. To further reach key audiences, the Consortium partnered with the FAO School Food Global Hub to cross-promote key publications, and with the Inter-Parliamentary Network for Education (IPNEd) to share useful research and guidance with parliamentarians (46). Every October, ahead of the SMC Ministerial Meetings and Global Summits, the Research Consortium holds its Annual Showcase, engaging a broad audience – including policymakers, academia, development agencies, and civil society – with the key findings that have emerged from across all of its global Communities of Practice. Held virtually over the course of three days, the Annual Showcase is an opportunity to hear from the researchers at the forefront of building the evidence base on what makes effective school meals policy and programming, as well as get an insight into the research priorities coming up. In 2024, the Consortium’s Annual Research Showcase drew an audience of more than 600 participants from 75 countries.

## Research Consortium 2024 Statement to the Ministerial Meeting of the School Meals Coalition

As previously described, the Research Consortium develops an Annual Research Statement which reports new programmatic and policy insights from the latest research on school meals and complementary interventions with the aim of increasing the reach, quality, and comprehensiveness of national programmes by 2030.

This section reproduces the Annual Research Statement developed by the Research Consortium for the 2024 SMC Ministerial Meeting hosted by the Government of Kenya. The 2024 Statement covers the accumulating and evolving programmatic and policy insights since the 2023 and 2022 Statements, which are included as Supplementary Annexes I, II to this manuscript, and concludes by suggesting priority areas of action for research directions to guide decision-making.

The Statement was prepared by the Research Consortium in support of the SMC member states. The insights consolidated within this Statement draw from analyses led by the Research Consortium’s global Communities of Practice with input from its Global Academy of more than 1,200 academics and practitioners.

## New evidence on the design and benefits of school meals programmes

### School meals are effective at improving growth, enrolment and learning, and in the case of educational outcomes, rank favorably relative to other popular education interventions

There is growing evidence that school meals positively impact education outcomes. Recent findings reviewing both experimental and quasi-experimental studies show that school meals can yield up to half a high-quality year of school per $100 spent. These programmes rank favorably relative to popular education programmes. These gains are mostly driven by studies with sizable effects on learning outcomes, although effects have high heterogeneity across settings. Effects appear to persist even in larger scale studies (17). These results appear to be supported by the very rigorous assessment procedures in both the gold-standard Cochrane Systematic Reviews that have been undertaken. The first Cochrane Systematic Review on this topic was conducted in 2007 and analyzed 18 studies that met the strict Cochrane criteria and found increased weight and height-for-age z-scores and modest benefits for math scores and intelligence test scores (18). These results were influential in catalyzing the increasing global roll out of school meals programmes over the first decade of the second millennium. The 2024 Cochrane Systematic Review has added to the depth and breadth of this analysis by increasing the number of admissible studies to 38 and the results to date provide further support for the 2007 observations (19).

### Recent reviews show the impact of cash transfers and school food as social safety net programmes, and suggest that both are effective and that neither is universally superior to meet the needs of vulnerable school-age children

School meals and cash transfers have long been the foundations of social protection programmes aimed at schoolchildren and adolescents. Both cash transfers and school meals aim to promote school participation while providing a safety net, but they do so in different ways. Cash transfers offer families direct financial support, while school meals provide in-kind assistance, ensuring that children receive at least one nutritious meal per day at school. In the last decade, both cash transfers and school meals have grown in global importance: cash transfers have become the default social assistance instrument for low-income countries, while school meals programmes have emerged as the world’s most extensive safety net and now serve about 41 percent of the world’s primary school children. The two programmes are often implemented together: for example, Brazil’s social reforms in the 2000s simultaneously introduced both the celebrated *Bolsa Familia* cash transfer programme and what is still the world’s second-largest universal, free school meals programme. Recent reviews of these two approaches show that neither is universally superior, as the relative effectiveness of cash transfers and food-based interventions like school meals is highly context-specific. In some cases, cash may offer greater flexibility and empowerment for families, while in others, food provision may be more effective in directly addressing hunger and nutritional deficiencies (20). Considering the complementary programmatic and policy strengths of each approach will be necessary when determining how best to achieve the goal of reaching the most vulnerable learners in primary and secondary schools in low- and lower-middle-income countries (21).

### School meals represent good value-for-money and, in our studies in Africa, are cost-beneficial in every region and sub-region where national analyses have been conducted

School meals deliver a wide array of multisectoral benefits, across at least the four sectors of: (1) education, via increasing enrolment, attendance and retention of learners in schools; (2) health and nutrition, via improving the nutritional status of learners and preventing certain diseases (e.g., anemia); (3) social protection, via providing a significant value transfer to households of the beneficiaries, through free or subsidized meals; and (4) local agricultural economy, via initiating and establishing stable markets for local smallholder farmers. They provide an integrated approach to improving outcomes in all of these areas, even if they are not necessarily the most efficient tool for any single domain on its own. By serving multiple purposes, school meals become an attractive policy choice, especially in resource-constrained environments where governments seek to maximize the impact of public programmes. Preliminary findings from eight countries in sub-Saharan Africa show that school meals programmes are cost-beneficial in every subregion of all eight countries, with respect to the gains obtained in the education and health and nutrition sectors. For every $1 invested in school feeding, benefits between $1 and $20 can be expected depending on the specific features of the national school meals programmes and on the local socioeconomic, educational and epidemiologic characteristics of each country. In some locations, these gains are higher for young girls than for young boys. School meals can bring large equity and redistribution benefits within countries to those most in need. Indeed, the value transfer (per school meal beneficiary) to households can be up to 10–20 percent of annual household food expenditures among the poorest.^3^

### Analysis of public policies in fifteen countries across income thresholds identifies common approaches that have helped ensure equitable coverage

The “Good Examples” Community of Practice aims to support national teams to draft case studies on national school meal programmes in all School Meals Coalition member states. Ultimately, a comparative analysis of all national programmes will help identify common challenges and their solutions. Case studies are currently underway in 48 countries. Analysis of the 15 studies published to date spanning four continents has highlighted common good practices, including: (i) utilizing nutritional standards to improve the quality of and diversity of school meals to support wellbeing; (ii) incorporating cultural practices within school menus as a mechanism to promote local purchase and national food sovereignty; (iii) involving students, parents, the community, and local authorities in the design and implementation of school meals programmes to foster ownership of the national programme; (iv) adopting a whole-school approach that goes beyond the provision of quality meals to also integrate complementary nutrition and health education to foster health promoting behaviors over the long-term; (v) in settings where school meals are not yet universally offered, some countries implement targeted budgeting for vulnerable children, with the objective of reducing poverty (for example, in Ethiopia and Benin); (vi) when numerous stakeholders (including international bodies) are involved in programme implementation, the creation of a national agency is key to coordinate efforts and ensure they adhere to public policies on nutritional standards and local sourcing by giving them agreements; and (vii) having emergency measures in place helps to ensure the continuity and resilience of school meals programmes, as has been done in the Ukraine (22–33).^4^

This result was the “3rd Priority” for research identified in the Consortium’s 2023 Annual Research Statement.

### Nutrition is important for the wellbeing of children and adolescents across the “first 8,000 days”

Recent research on the “first 8,000 days” of life, which spans the development period from conception to the beginning of adulthood, underscores the critical importance of nutrition in shaping lifelong health outcomes. During this period, optimal nutrition plays a pivotal role in physical growth, behavioral and cognitive development, and the prevention of chronic and infectious diseases. Research has emphasized the importance of maternal nutrition during pregnancy in influencing growth and long-term health, with poor maternal nutrition linked to adverse outcomes such as low birth weight and stunted growth, which can persist into adulthood. Additionally, early childhood nutrition, particularly during the first 1,000 days of life (from conception to two years of age), has been highlighted as crucial for brain development and immune system function. Studies have demonstrated that adequate intake of key nutrients during this period is essential for neurodevelopment and reducing the risk of cognitive impairments later in life. Nutrition during middle childhood and adolescence, the “next 7,000 days,” is increasingly recognized for its potential role in supporting catch-up growth during periods of rapid growth that occur between ages 5–9 years and at puberty, the brain rewiring that occurs throughout this period and is most intense in later adolescence and, when combined with appropriate education, can help the establishment of lifelong healthy behaviors including dietary preferences. Good nutrition during these critical periods of development covering the next 7,000 days will help set the stage for long-term health including reduced risk for both non-communicable and infectious diseases, improved cognitive and work performance, improved reproductive health, and life expectancy. Altogether, recent research continues to highlight the foundational role that nutrition plays throughout the “first 8,000 days” in promoting long-term health, emphasizing the need for comprehensive strategies to ensure adequate nutrition throughout this development phase (34). The world currently invests 2.8 trillion USD annually in education during the next 7,000 days, yet estimates suggest that current investment in well-being during this phase represent less than 2 percent of the amount invested in education.

This result was the 2nd priority identified for further research in the Consortium’s 2023 Annual Research Statement.

## New evidence on effective school meals policies

### Prolonged school closures in response to the COVID-19 pandemic had strong negative social, educational and economic consequences, and have highlighted the value of school-based programmes for the wellbeing of children and young people

The consequences of school closures during the COVID-19 pandemic were recently the subject of the World Bank Group’s influential analyses to identify Disease Control Priorities. The decision to close schools at the onset of the pandemic reflected public health experience with managing influenza transmission. This precedent in turn led countries worldwide to close schools as a precautionary measure, even before direct evidence of the epidemiological role of children and adolescents in the transmission of COVID-19 was understood. The emerging evidence for public health impact of these decisions remains mixed, however, there is generally low confidence in the evidence that closing schools, or preventative actions in schools which were not closed, had meaningful consequences for the transmission of COVID-19 for either the primary school population or the general population. In contrast, global evidence shows considerable consequences for human capital formation and wellbeing of learners; school closures in the context of the COVID-19 pandemic led to a 9 percent reduction in learning achievement in standardized tests, which, over time, are estimated to negatively affect lifetime earnings by 6 percent. The closures had additional unforeseen societal consequences, including increased rates of early marriage and early pregnancy for school-age girls, of inappropriate labor for all school-age children, and substantial and often irreversible drop-out from school. The counterfactual experience of closing schools, and removing most forms of support to schoolchildren and adolescents, has spurred national governments to re-establish and strengthen investments in school-based services. National school meals programmes in particular have proven to be important in elevating school participation and attendance, especially important given that chronic absenteeism has gone up after the pandemic-driven closures. Lessons learned from this pandemic can shape government actions in schools in subsequent pandemics, which will again need to weigh the trade-offs between protecting public health and the integrity of the school system. The creation of the School Meals Coalition is highlighted as a particularly positive action (14).

### The World Bank Group’s Systems Approach for Better Education Results (SABER) has become an institutionalized policy instrument in low- and lower-middle-income countries

Since its introduction in 2011, at least 59 countries have used these tools 81 times to self-evaluate their national policies for school meals and complementary programmes against international benchmarks and to help identify actionable priorities to strengthen national programmes. Globally the SABER School Feeding tool has been adopted by 68 percent of the world’s low-income countries and 54 percent of lower-middle-income countries. SABER is unique in that this is a government-led, government-completed process. An analysis of SABER surveys suggests that countries with longer established national school meals frameworks tend also to be more advanced in other policy areas, and that the weakest policy areas relate to programme design, implementation and fiscal space (35). Given the complementarities between school meals and other school-based health interventions, the World Bank Group and WFP have combined key elements of the SABER School Feeding and SABER School Health framework into a single, comprehensive policy tool. ‘Healthy-SABER’ is envisaged to further engage multisectoral actors in the design of effective and holistic school health policies and clarify key areas for further investment, and is currently being rolled out by governments across Africa, with support from the Human Development Sectors of the World Bank Group and WFP.

This result was the 4th priority identified for further research in the Consortium’s 2023 Annual Research Statement.

### National governments have demonstrated a commitment to adopting planet-friendly school meals policies, and modeling data estimates that implementing these commitments will have significant positive impacts on human and planetary health

The white paper on *School Meals and Food Systems: Rethinking the consequences for climate, environment, biodiversity and food sovereignty*, was presented on the main stage at COP 28 in Dubai in December 2023. This paper was co-written in collaboration with more than 160 authors from 87 global organizations and explores how school meals programmes can help deliver improved health, environmental, climate and economic outcomes. Governments typically hold the policy levers for their national school meals programmes. These programmes represent the majority (around 70 percent) of all publicly managed food systems, and globally reach 418 million children daily. They therefore provide a unique opportunity for government policy to create significant change in food practices, at scale. The White Paper identified priority areas relating to food use that could be addressed by policy levers immediately available to national programmes: planet-friendly menu changes, clean and energy efficient cooking solutions, reduction of food waste, holistic food education, and procurement policies that create demand for ecological and fair agricultural production. Modeling data estimate that adopting planet-friendly menus and reducing food waste, could reduce mortality from dietary risks by 10–20 percent, with a 50–60 percent reduction in environmental impacts (including greenhouse gas emissions, land use, freshwater use and eutrophication), and lift 120 million people out of undernourishment. In addition, it is estimated that these effects can be carried into adulthood, potentially resulting in a 12–20 percent reduction in deaths (or 3 million fewer deaths) (36). The findings in this report have subsequently been widely endorsed in high impact scientific journals, and are being explored and tracked in practice by several countries, including Kenya, Norway, Rwanda and Uganda. A specific need has been identified to create a toolkit that can be used by governments to assess the benefits and costs of modifying their national programmes.^5^

This result addresses the 1st priority identified for further research in the Consortium’s 2023 Annual Research Statement.

### Procurement practices have the potential to influence farming practices, including driving a renewed focus on regenerative agriculture

The planet-friendly White Paper identified the role of procurement in contributing to changes in farming practices over the long run. However, the follow-up research suggests that school meals can contribute to wider food system transformation through multiple pathways and processes, besides the more traditionally understood pathway of output support for food producers. Through its various operational components such as menu development and school gardens, it provides a unique platform for programme and policy engagement across public health, conservation and food production. Very importantly, given the localized nature of most school meals programmes food system transformation catalyzed through school meal programmes is particularly context sensitive and community driven (9). There are now five country-specific studies across sub-Saharan Africa which are exploring these issues including developing appropriate interdisciplinary research methods to capture the diverse element of interactions between school meals programmes and menus and the broader-food system. Some key focus areas of the current research include the role of school meals in promoting climate smart foods, improving gender equity, access to financial resources and supporting agrobiodiversity. The research also explores how school meal programmes can help align policies around issues such as farmer sharing of planting material and adoption of biofortified foods, in the context of traditional farming practices and food sovereignty. A specific research strategy is also being developed to understand how this transformation can also influence agricultural methods, such as promoting regenerative agriculture, and what are the associated drivers, constraints and risks.

This result addresses the 1st priority identified for further research in the Consortium’s 2023 Annual Research Statement.

## Priority actions going forward to support the School Meals Coalition member states

Based on the evidence accumulated over three years of analysis with the member states of the School Meals Coalition, and in addition to the ongoing research programmes of the Communities of Practice and insights from our Global Academy, the Research Consortium for School Health and Nutrition has prioritized the following five research topics for the coming year:

1. **Support government action to enhance the climate and environmental sustainability of national school meals programmes**. Work with governments to create an open access “sustainability toolkit” of policy and programmatic tools to assist countries with the design or adaptation of school meal programmes that optimize co-benefits for people and the planet. These tools would aim to strengthen the ability of governments to predict the health and resilience outcome of different policy choices, and contribute to evidence-based decisions that are reflective of the local context.
2. **Support the roll-out of the School Meals Coalition’s Data and Monitoring Initiative to improve the availability of data on school meals programmes**. Effort in this area will include the creation and data-population of a global database for school meals and school health, and analyses to better identify actionable responses to challenges to equity, coverage and programme quality.
3. **Support the roll-out of the pilot Healthy-SABER tool as a joint action with WFP and the World Bank.** The Consortium will build on its role of analyzing the effectiveness of the various tools since their launch in 2011 to also include a focus on institutionalizing the role of Healthy-SABER as a policy monitoring tool and identifying a permanent, open-access policy archive.
4. **Explore the options for professionalizing training in school meals programming.** This will include scoping the current availability of formal and informal, on-site and distance training opportunities for school food professionals.
5. **Share policy information on the value-for-money of school meals programmes.** There is now a substantial and growing portfolio of evidence on the multisectoral returns from school meals programmes, and it is now possible to distill the implications of these findings for policy-makers, with a focus on the returns to nutrition/health, education, social protection and agriculture. There is also a need to undertake two types of systematic reviews: one of programme costs, to update the current most widely cited dataset which pre-dates the 2008 financial crisis; and the second of the reported costs, effectiveness, and cost-effectiveness of school meals and school health programmes. Finally, there is much to be learnt from the well documented performance of the largest and most mature school meals programmes across the globe, including the programmes in Brazil, Finland, India, Japan, Republic of Korea, South Africa and Sweden. The aim is to provide government decision makers with authoritative data on the scale of return from investing in school meals programmes, by providing quantitative estimates to support policy decisions.

## Conclusion

The Research Consortium develops Annual Research Statements ahead of the School Meals Coalition Global Summits and Ministerial Meetings as part of its commitment to assemble and share policy relevant evidence with the (now) 109 member states. These Annual Research Statements consolidate the latest insights on school meals as they relate to policy and programmatic design and to the evidence of effectiveness and cost-efficiency, and conclude with research priorities for the forthcoming year. These insights draw upon the formative work of the Consortium’s Communities of Practice and from its Global Academy of more than 1,200 professors, scholars, and practitioners from more than 110 countries.

## Footnotes

^1^The six regional bodies that have joined the School Meals Coalition include: the African Union, Community of Portuguese Language Countries, Economic Community of West African States, European Commission, Organization of Eastern Caribbean States, and the Southern African Development Community.

^2^Thematic research requests are identified through bilateral requests, through discussions that emerge from bi-monthly meetings with the SMC Task Force and Working Group, from priorities that were agreed at regional SMC convenings, and with input from the Consortium’s Global Academy of more than 1,200 members.

^3^Since the publication of the 2024 Annual Research Statement, value-for-money studies have been validated in: Burundi (37), Ethiopia (38), Malawi (39), and Namibia (40).

^4^Since the publication of the 2024 Annual Research Statement, five additional country case studies have been finalized: Burundi (41), Ecuador (42), Kenya (43), Morocco (44), and Nigeria (44).

^5^Since the publication of the 2024 Annual Research Statement, the Planet-Friendly Diets Community of Practice published a related commentary on the opportunities for planet-friendly school meals to improve children’s health and leverage change in food systems in The *Lancet* Planetary Health (36).

## Group members of Research Consortium for School Health and Nutrition

Donald A.P. Bundy, London School of Hygiene and Tropical Medicine, UK; Linda Schultz, London School of Hygiene and Tropical Medicine, UK; Kate Morris, London School of Hygiene and Tropical Medicine, UK; Jasmine Catmull, London School of Hygiene and Tropical Medicine, UK; Georgia Crowley, London School of Hygiene and Tropical Medicine, UK; Sarah Vickers, London School of Hygiene and Tropical Medicine, UK; Patricia Eustachio Colombo, London School of Hygiene and Tropical Medicine, UK and Karolinska Institutet Elizabeth Kristjansson, University of Ottawa, Canada; Muna Osman, University of Ottawa, Canada; Gilbert Miki, London School of Hygiene and Tropical Medicine, UK; Edward Makumbe, African Union Commission, Zimbabwe; Noam Angrist, What Works Hub for Education, University of Oxford, UK; Stéphane Verguet, Harvard T.H. Chan School of Public Health, USA; Sylvie Avallone, L’Institut Agro, France; Heli Kuusipalo, National Institue for Health and Welfare, Finland; Aurélie Fernandez, Independent Consultant Robert Akparibo, University of Sheffield, UK; R. Gopinath, M.S. Swaminathan Research Foundation, India; Abimbola Adesanmi, Partnership for Child Development, Nigeria; Samrat Singh, Imperial College London, UK; Jean D’Amour Manirere, University of Rwanda, Rwanda; Silvia Pastorino, London School of Hygiene and Tropical Medicine, UK; Jacqueline Kungú, African Population and Health Research Center, Kenya; Dirce Maria Lobo Marchioni, University of São Paulo, Brazil; Semiramis Domene, University of São Paulo, Brazil.
