# Supplementary material for: Policy insights for national school meals programmes: Annual Research Statements to the members of the School Meals Coalition - 2022, 2023, and 2024
Source: Front Public Health. 2025 Jul 18;13:1416165. doi: 10.3389/fpubh.2025.1416165 (PMC12315696; doi:10.3389/fpubh.2025.1416165)
Supplement: Supplementary file 1 [file Table_1.docx]

Supplementary Material

**Statement to the Ministerial Meeting of the School Meals Coalition**

*Prepared by the Research Consortium for School Health and Nutrition,*

*an initiative of the School Meals Coalition*

18 October 2022

The Research Consortium for School Health and Nutrition was launched as a global initiative in 2021 to provide independent, evidence-based guidance to the more than 70 member states of the School Meals Coalition as they recover from the COVID-19 pandemic and rebuild the health, education and future of their schoolchildren and adolescents.

For the last 12 months researchers from across the world have co-created global Communities of Practice to examine the impact of the pandemic and to identify the key lessons learned to support recovery.

This Statement summarizes the key lessons learned up to October 2022, and suggests the implications for practice. The Statement was prepared by the Research Consortium on behalf of the School Meals Coalition for the parliamentarians and public-policymakers of the member countries of the Coalition.

**Overview**

**Key Messages on What We Have Learned from the Pandemic**

1. The school closures of the pandemic have caused major, long-term harm in all countries.
2. The Global Food Crisis is making recovery more difficult, and even more necessary.
3. Recovery of the education system and investment in young people is crucial for the future of this generation and the Wealth of Nations.
4. The well-being, education and development of young people during the first 8,000 days of life are crucial for the economic and social future of all countries, whether high- or low-income countries.

**Lessons on the Design and Benefits of Programmes**

1. Benefits for education: more children go to school, and they learn better while there.
2. Benefits for health and nutrition: children and young people enjoy better well-being and learning, and establish life-long health and dietary practices which promote their well-being, productivity and longevity as adults.
3. Benefits for social protection: national school meals programmes are among the most extensive and effective safety nets in the world.
4. Benefits for diet and food systems: promoting healthy diets, providing stable markets, and encouraging agricultural practices that support biodiversity, food sovereignty and climatic resilience.

**Lessons from National Programmes**

1. National school meals programmes are exceptionally good examples of self-reliance, they are almost entirely supported by domestic funds.
2. Graduation to middle-income status presents a key opportunity for the transition to self-reliance.
3. There are 73 million especially vulnerable children that have never been reached by school meals programmes.
4. The pandemic has affected every child on earth, and rebuilding requires action at the same enormous scale as the challenge.

**Recommendations Going Forward**

1. Recognize that the well-being of the learner is essential to educational attainment, and prioritize national school meal programmes and complementary school health interventions as essential counterparts to educational investments, especially at the Transforming Education Summit 2022 and at the Summit for the Future 2023;
2. Scale-up and extend the coverage of national programmes to ensure that these safety nets are in place to protect all vulnerable school children as an essential response to the Global Food Crisis;
3. Strengthen monitoring of the school health agenda by working with UNESCO to ensure that school meal and complementary school health programmes are included explicitly in the SDG4 framework; and
4. Encourage a more robust donor response to match the substantial domestic commitments already being made by governments, especially a dialogue with international financing institutions IFIs to generate a financing response for the most needed and most at-risk programmes in low-income countries.

**Key Messages on What We Have Learned from the Pandemic**

**The pandemic has affected all the countries in the world, irrespective of income level. In building back from the pandemic, the following four key messages are particularly important in deciding upon national policy for development and reconstruction.**

1. **The school closures of the pandemic have caused major, long-term harm in all countries**

The school closures to try and arrest the transmission of COVID-19 resulted in 1.5 billion children in 199 countries being out of school. This is the first time since adoption of formal, universal education that we have seen the effects of a world without an education system. This counterfactual showed that home-schooling, distance education and other EdTech alternatives cannot substitute for traditional school systems. It also showed that the school system is key to supporting the well-being and health of school-age children and adolescents, through delivering nutritious food and health interventions, and that the absence of the safe environment of the schools has detrimental social consequences, increasing the risks of child abuse, early pregnancy and inappropriate labour**.** The pandemic has worsened a pre-existing learning crisis such that today the World Bank estimates that 70% of children under the age of 10 years in low-income countries cannot read or write a simple sentence. Restoring and strengthening the school system should be a major priority for all nations, irrespective of income level.

1. **The Global Food Crisis is making recovery more difficult, and even more necessary**

Hardly had schools started to reopen when the world rolled into a new crisis of food availability and affordability, in part driven by the war in Ukraine. New data from the World Food Programme (WFP), the Research Consortium for School Health and Nutrition, and the Education Commission estimates that 153 million children under 18 are acutely food insecure, nearly half of the 345 million people worst hit by rising global hunger. Data also shows that these figures have been rising over the past two years, leading to 23 million more acutely food insecure children now than in 2020, before the pandemic. School-age children are bearing the brunt of today’s Global Food Crisis with far reaching consequences for children, communities, and countries for decades to come. It is an important additional benefit of national school meals programmes in 2022 and beyond that they help countries respond to the Global Food Crisis.

1. **Recovery of the education system and investment in young people is crucial for the future of this generation and the Wealth of Nations**

Good health, well-being and education during childhood and adolescence are crucial to an individual achieving their full potential, and to the creation of Human Capital for a nation. It is because nations recognize the value of educating their children that free, compulsory basic education is a near-universal global priority, and many countries are now extending this investment to include secondary education. The consequences of school closures during the pandemic have taught us that there is no effective substitute for school systems to take care of the education, well-being and health of children during the crucial first 8,000 days of life (birth to the early twenties), and to help young people realize their potential as individuals, and provide the community with the skills, good health and longevity that are central to Human Capital. Evidence from the World Bank shows that countries that effectively create Human Capital can expect their population to contribute some 70% to 80% of the nation’s wealth, while for those who do not, this share is as low as 30% to 40%.

1. **The well-being, education and development of young people during the first 8,000 days of life are crucial for the economic and social future of all countries, whether high- or low-income countries**

Creating human capital is essential for the development of all countries but for different reasons. In high-income countries with aging populations where the median age is around 40 years (e.g. Europe, USA, Japan), school-age children and adolescents represent less than 10% of the total population, yet it is this small population which will ultimately be relied upon to generate 70% of the wealth for all. Rich, aging populations clearly need to sustain and strengthen investment in these young people. In low-income countries with young populations, where the median age can be as low as 15 to 16 years (e.g. Niger, Chad, Sierra Leone), some 50% of the population is in the 8,000 days age range and yet will contribute only some 30% to 40% of the nation’s wealth. Furthermore, in the continuing absence of a demographic transition, the proportion of youth will continue to rise, and increase dependency. For all countries, irrespective of income level, recovery from the pandemic and re-establishing the benefits of the school system will be crucial to national development.

**Lessons on the Design and Benefits of Programmes**

**Properly designed school meals and complementary school health programmes are key to the investment in young people. Well-designed programmes that improve the well-being and education of young people during the first 8,000 days of life can offer returns of $9 (range $7 to $35) for each $1 invested. Supplementary Figure 1 shows that this is a cumulative return based on the sum of the multiple returns to different sectors. In this section we explore the lessons learned for four important sectors, and ask the same question for each sector: what are the benefits of school meals and complementary school health programmes?**

Supplementary Figure 1. School feeding achieves high returns on investment across at least four sectors: (i) social protection; (ii) education; (iii) agriculture; and (iv) health and nutrition.


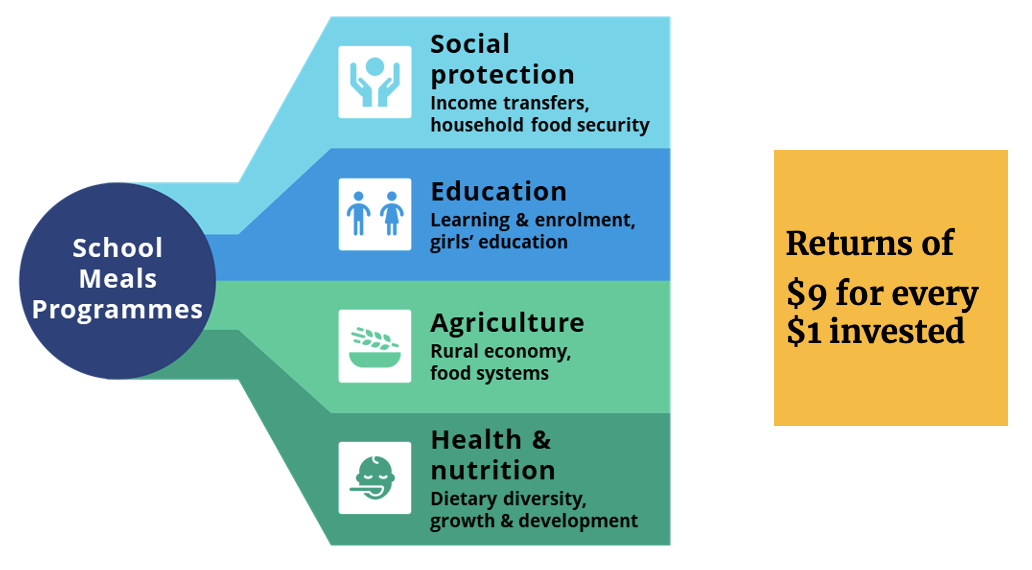


*Source: Adapted from Verguet et al. 2020*

1. **Benefits for education: more children go to school, and they learn better while there**

Providing school meals is an effective tool for promoting access to education with a typical sustained increase of around 9% participation, and rates much higher in countries where girls’ participation in education has traditionally been low. Supplementary Figure 2 shows, using the new metric of Learning-Adjusted Years of Schooling (LAYS) which combines both quantity and quality of education received, that improving the health and well-being of the learner has benefits for learning. Importantly, the current evidence suggests that the scale of improvement in learning from school meals and school health is of a similar order of magnitude to interventions which we already know to be effective, such as those promoting early child development, better qualified teachers and better classroom practices. If countries invest in these traditional areas to improve the supply of schooling and learning, then it also makes good sense to invest in the demand side through investing in the health and well-being of the learner.

Supplementary Figure 2. School meals and complementary school health interventions offer good value for money


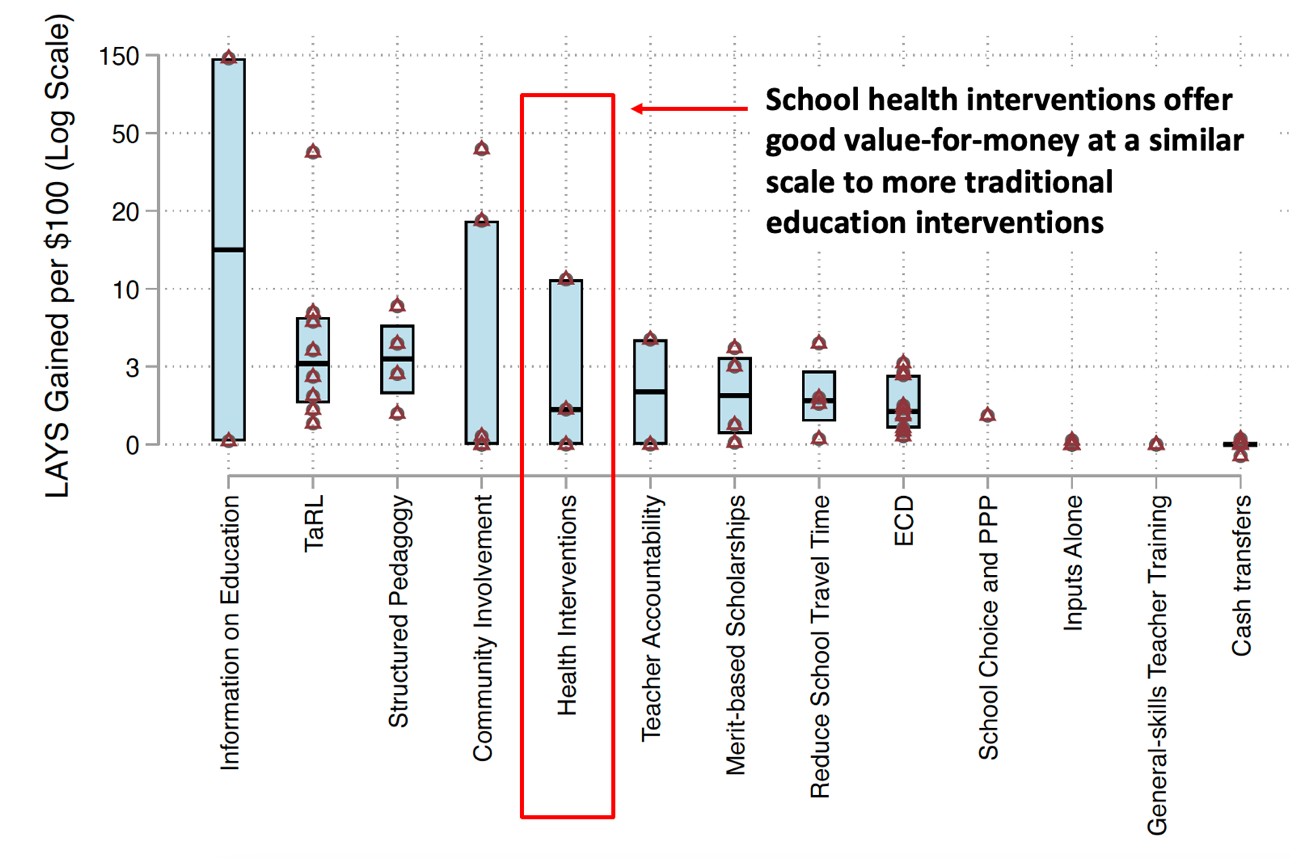


*Source: Adapted from World Bank Policy Research Working Paper No. 9450*

1. **Benefits for health and nutrition: children and young people enjoy better well-being and learning, and establish life-long health and dietary practices which promote their well-being, productivity and longevity as adults**

We have already explained how well-designed school meals and complementary school health programmes can contribute to children’s learning by improving their health and well-being when they are in school and being educated. But these are dynamic benefits such that good health, nutrition, fitness and mental health developed during the school years can lead to a long and healthy life. Comprehensive school health programmes which help establish life-long good health, dietary and exercise practices, are amongst the most effective ways of reversing the growing epidemics of obesity and non-communicable diseases in later life. In addition to making young people healthier, these interventions have the potential to significantly reduce the future costs and demands on health systems for adults. These returns are important for all countries and all income levels.

1. **Benefits for social protection: national school meals programmes are among the most extensive and effective safety nets in the world**

The response to the Fuel, Food and Financial Crises of 2008 included a substantial increase in the coverage of school meals and complementary school health programmes in both high- and low-income countries, and was recognized to be an immediate and effective way of reaching directly to young people, becoming the most extensive social safety net in the world. The school closures of COVID-19 effectively prevented these programmes from playing this supportive role in 2020, and instead undermined the social safety nets. Attempts to replace the programmes with cash transfers had limited success but have spotlighted some key advantages of school meals and complementary school health programmes over cash transfers: they benefit the target child directly; they offer subsidiary benefits for education, nutrition and well-being; and they stimulate the local agricultural and service economies. These benefits are observed in countries at all income levels, indeed the evidence for the value of school meals as safety nets in high-income countries is particularly strong (e.g. sustaining programmes during vacation periods). Countries lost all these potential benefits when they closed schools during the pandemic, and so restoring, protecting, and scaling up these programmes now can make a major contribution to the national safety net, and an important part of the response to the food crisis.

1. **Benefits for diet and food systems: promoting healthy diets, providing stable markets, and encouraging agricultural practices that support biodiversity, food sovereignty and climatic resilience**

Effective school meals programmes depend on effective food systems, which in turn depend on effective agriculture. Too often the focus has been on the quantity and not the quality of food, yet the Global Education Forum 2022 estimates that more than 3 billion people have low quality diets. School meals programmes can help address this. At the same time, the programmes provide stable, predictable markets for local farmers who can then focus on local production, including micronutrient dense crops targeted at growing children, and supporting local cultural preferences and climatic realities. This is a virtuous cycle that can help local economies thrive and promote food sovereignty, while encouraging local agrobiodiversity which confers climate resilience. Linking local agriculture to national school meals programmes is a proven approach that is very successful in many countries, but there is major scope for further adoption and development.

**Lessons from National Programmes**

**There are important lessons to be learned from the global experiences of financing and implementing national programmes. The following 4 messages spotlight some of the most important learnings.**

1. **National school meals programmes are exceptionally good examples of self-reliance, they are almost entirely supported by domestic funds**

In 2020, before the pandemic, more than 90% of the funds used to support national school meals programmes worldwide arose from domestic budgets. The largest universal and free programmes, for example in India feeding some 90 million daily and Brazil some 40 million, rely entirely on local resources. The most successful programmes are intimately linked to local agricultural production and take full advantage of the virtuous cycle of local procurement to invest in the next generation. Self-reliance is the norm in high-income, higher-middle-income and lower-middle-income countries, and countries at these income levels have created self-sustaining programmes that support local economies and help build human capital. But food does have a global floor price which is beyond some low-income countries, although even here nearly a third of costs on average are met from domestic funds. For most countries school meals programmes are a sustainable investment, and most programmes are, or can become, self-reliant. The major role for external investment is to help middle-income countries transition to self-reliance, and to provide interim support to help low-income countries to create the human capital needed to strengthen their economic growth.

1. **Graduation to middle-income status presents a key opportunity for the transition to self-reliance**

More than 44 countries have transitioned from reliance on external funds to domestically supported programmes in the last 20 years. This transition occurs most frequently when countries cross the threshold from low-income to lower-middle-income status because the growing economy presents the opportunity to break through the price floor and afford the cost of food. The price floor exists because there is a global minimum cost of food: in high income countries school food is less than 1% of GDP per capita, while in low-income countries it represents 8% or more. The comparison is more stark with the cost of education: in low-income countries school meals are on average 96% of the per capita education budget, and often much more. When countries graduate to lower-middle-income status this proportion falls to 42%, and at middle-income status to less than 11%. As the economy strengthens, the cost of food becomes increasingly affordable. Longitudinal analysis of the increase in school meals coverage in Africa between 2013 and 2020, showed that the fastest rate of growth in coverage was in lower-middle-income countries at the time that they graduated from low-income status. Supporting countries in making that transition is a particularly effective use of external funds.

1. **There are 73 million especially vulnerable children that have never been reached by school meals programmes**

Even before the pandemic, about 73 million school-age children, in 60 countries, living in extreme poverty and hunger did not have access to school meals programmes. The member countries of the School Meals Coalition have identified reaching these children as a priority, which is set out as the Second Goal of the Coalition. The annual cost of reaching these children with school meals and complementary school health interventions is estimated at US$5.8 billion, with about half that amount for low-income countries.

1. **The pandemic has affected every child on earth, and rebuilding requires action at the same enormous scale as the challenge**

The Research Consortium for School Health and Nutrition was established by the School Meals Coalition to provide parliamentarians, policymakers and programme managers with independent, evidence-based guidance on recovering from the COVID-19 pandemic. This implies a global response on the scale of hundreds of millions of children. To make this possible, the Research Consortium has sought to minimize its governance structure (it is coordinated out of a small Secretariat in the London School of Hygiene and Tropical Medicine) and invest in creating a network from the existing global networks working on school health, and to leverage the established reach and expertise of academics and practitioners worldwide. These global Communities of Practice are essential engines of research, analysis, and experience and are strongly aligned with and led by expertise from the Coalition countries. The value of school meals for the well-being of children can be multiplied by complementary school-based health interventions, so we are also working actively to coordinate with other multi-country networks that support other activities critical to the health, mobility, and cognitive development of the learner. For example, the END Fund and the Global Schistosomiasis Alliance to address neglected tropical diseases, AMREF to address malaria, the International Agency for the Prevention of Blindness for eye-health and vision, and the International Society for Physical Activity and Health (ISPAH) to support the physical health of schoolchildren.

**Recommendations Going Forward**

There has been remarkable progress in this initial year of the School Meals Coalition, but there is no time for complacency. The bleak global economic outlook and the growing constraints on fiscal space make recovery both more essential and more difficult. Based on the evidence available after one year of analysis, the Research Consortium for School Health and Nutrition calls upon the 73 member states of the School Meals Coalition to consider supporting four priority areas of policy change as we move forward:

1. **Recognize that the well-being of the learner** **is essential to educational attainment**, and prioritize national school meal programmes and complementary school health interventions as essential counterparts to educational investments, especially at the Transforming Education Summit 2022, SDG Summit 2023, and at the Summit for the Future 2024;
2. **Scale-up and extend the coverage of national programmes** to ensure that these safety nets are in place to protect all vulnerable school children as an essential response to the Global Food Crisis;
3. **Strengthen monitoring of the school health agenda** by working with UNESCO to ensure that school meal and complementary school health programmes are included explicitly in the SDG4 framework; and
4. **Encourage a more robust donor response** to match the substantial domestic commitments already being made by governments, especially a dialogue with international financing institutions to generate a financing response for the most needed and most at-risk programmes in low-income countries.

**Acknowledgements:** This Statement was prepared by the Research Consortium for School Health and Nutrition as part of its commitment to the School Meals Coalition. It was conceived and written by the members of the Research Consortium Secretariat (Donald Bundy, Linda Schultz, Kate Morris, Samantha Owen and Mary-Alice McDevitt) who are entirely responsible for the content. We would like to thank the following members of the Research Consortium for valuable discussions in the preparation of this Statement: Noam Angrist, Sylvie Avallone, Myles Bremner, Mary Brennan, Sinead Brophy, Greta Defeyter, Sinead Furey, Bibi Giyose, Anouk Gouvras, Peter Hangoma, Jayne Jones, Elizabeth Kristjansson, Carol Karutu, Heli Kuusipalo, Sam Mayer, John McKendrick, Gabriella McLoughlin, Ole Norheim, Muna Osman, David Rollinson, Samrat Singh, Stéphane Verguet, Johannes Waltz, Kevin Watkins, Jayne Woodside, Katherine Woolley and Francis Zotor.

**Key References**

**Key Messages on What We Have Learned from the Pandemic**

1. Research Consortium for School Health and Nutrition and the Sustainable Financing Initiative. The Investment Case for School Health and Nutrition: Memo #4 to inform the Global Education Forum. April 2022. Available at: https://www.docdroid.net/GJLq1sN/the-investment-case-for-school-health-and-nutrition-7-april-2022-final-pdf
2. Bundy DAP, de Silva N, Horton S, Jamison DT and Patton GC. 2017. Child and Adolescent Health and Development (with a Foreword by Gordon Brown). Volume 8. In DT Jamison, R Nugent, H Gelband, S Horton, P Jha, R Laxminarayan and C Mock, eds. Disease Control Priorities (3rd edition). Washington, DC, World Bank.
3. Lange GM, Wodon Q, and Carey K. (2018). “The Changing Wealth of Nations 2018: Building a Sustainable Future.” The World Bank.
4. CGD. 2022. Schooling for All: Feasible Strategies to Achieve Universal Education. CGD: Washington. Available at: https://www.cgdev.org/sites/default/files/schooling-for-all-feasible-strategies-universal-eduction.pdf

**Lessons on the Design and Benefits of Programmes**

1. Verguet S, Limasalle P, Chakrabarti A, Husain A, Burbano C, Drake L, Bundy DAP. The Broader Economic Value of School Feeding Programs in Low- and Middle-Income Countries: Estimating the Multi-Sectoral Returns to Public Health, Human Capital, Social Protection, and the Local Economy. Front Public Health. 2020; 8:587046.
2. Angrist N, Evans DK, Filmer D, Glennerster R, Rogers FH, Sabarwal S. 2020. How to Improve Education Outcomes Most Efficiently? A Comparison of 150 Interventions Using the New Learning-Adjusted Years of Schooling Metric. Policy Research Working Paper; No. 9450. World Bank, Washington, DC. © World Bank. https://openknowledge.worldbank.org/handle/10986/34658 License: CC BY 3.0 IGO.
3. Angrist N, Djankov S, Goldberg PK, and Patrinos HA. "Measuring human capital using global learning data." Nature 592, no. 7854 (2021): 403-408.
4. Bundy DAP, Burbano C, Grosh M, Gelli A, Jukes M, Drake L. 2009. Re-thinking School Feeding: Social Safety Nets, Child Development, and the Education Sector. Directions in Human Development. Washington, DC, World Bank Group.
5. WFP, FAO, IFAD, NEPAD, GCNF and PCD. 2018. Home-Grown School Feeding Resource Framework. Technical Document. Rome. Available at: https://www.wfp.org/content/home-grown-school-feeding-resource-framework

**Lessons from National Programmes**

1. World Food Programme. State of School Feeding Worldwide 2020. Rome (Italy): World Food Programme (WFP).
2. Drake LJ, Lazrak N, Fernandes M, Chu K, Singh S, Ryckembusch D, Nourozi S, Bundy DAP, Burbano C. Establishing Global School Feeding Program Targets: How Many Poor Children Globally Should Be Prioritized, and What Would Be the Cost of Implementation? Front Public Health. 2020 Dec 2;8:530176.
3. Drake L, Fernandes M, Aurino E, Kiamba J, Giyose B, Burbano C, Alderman H, Mai L, Mitchell A, and Gelli A. 2018. School Feeding Programs in Middle Childhood and Adolescence. In D.A.P. Bundy, N. de Silva, S. Horton, D.T. Jamison and G.C. Patton, eds. Re-Imagining School Feeding: A High-Return Investment in Human Capital and Local Economies. Washington, DC, World Bank.
4. Bundy D, Burbano C, Gelli A, Risley C, Neeser K. (2011). On the transition to sustainability: An analysis of the costs of school feeding compared with the costs of primary education. Food and Nutrition Bulletin, 32(3): 201-5.
5. Kristjansson EA, Gelli A, Welch V, Greenhalgh T, Liberato S, Francis D, Espejo F. 2016. Cost and cost-outcome of school feeding programmes and feeding programmes for young children: evidence and recommendations. International Journal of Educational Development. 48: 79-83.
6. The Economist. Should every schoolchild eat free? Sept 1 2022. Available at: <https://www.economist.com/international/2022/09/01/should-every-schoolchild-eat-free>
