# Supplementary material for: Policy insights for national school meals programmes: Annual Research Statements to the members of the School Meals Coalition - 2022, 2023, and 2024
Source: Front Public Health. 2025 Jul 18;13:1416165. doi: 10.3389/fpubh.2025.1416165 (PMC12315696; doi:10.3389/fpubh.2025.1416165)
Supplement: Supplementary file 2 [file Table_2.docx]

Supplementary Material

**Statement to the Ministerial Meeting of the School Meals Coalition**

*Prepared by the Research Consortium for School Health and Nutrition,*

*an initiative of the School Meals Coalition*

18 October 2023

The Research Consortium for School Health and Nutrition was launched as a global initiative in 2021 to provide independent, evidence-based guidance to the 90 member states of the School Meals Coalition as they recover from the COVID-19 pandemic and rebuild the health, education and future of their schoolchildren and adolescents.

This Annual Research Statement reports new programmatic and policy insights in 2023 as well as accumulating and evolving understanding of the research in this area since the 2022 Ministerial Meeting. The Statement concludes by suggesting the implications for policy decisions, with the aim of increasing the reach, quality, and comprehensiveness of national school meals programmes by 2030.

The Statement was prepared by the Research Consortium on behalf of the School Meals Coalition for the parliamentarians and policymakers of the member countries of the Coalition. The insights consolidated within this Statement draw from analyses led by the Research Consortium’s global Communities of Practice, as indicated under each statement below, including by the early career researchers and young scientists.

## New Insights into the Design and Benefits of Programmes

*School closures during the COVID-19 pandemic continue to have a powerful influence on the momentum of school meal programmes.*

The school closures from the COVID-19 pandemic continue to cast a shadow worldwide on education outcomes and the prospects of the affected generation of school children. Some children, especially girls, have dropped out permanently; others have become persistently absent – up to 20 percent even in some high-income countries – while continuing inconsistency in marking examinations is affecting participation in tertiary education. The universality of this effect has spurred the growth of the SMC over the past two years, bringing the Coalition to (now) 97 member states and more than 100 partner organizations. At the Food Systems Stocktaking +2 event in Rome, the SMC was recognized as the most substantial Coalition to arise from the 2021 Food Systems Summit. This momentum is also confirmed by the rebound in coverage of programmes: today, 418 million children are receiving a daily meal in school, exceeding levels prior to the COVID-19 pandemic in high- and middle-income countries and even some low-resource countries, notably in Benin, Honduras and Rwanda (1). This success suggests the Coalition has largely achieved its first goal, but the global average of one-in-two school-going children receiving a meal masks the difference among high-income countries (with 60 percent coverage) and low-income countries (20 percent).

*Universal school meals are fast becoming the most popular programming approach.*

Today, 186 million children receive school meals through universal programmes – more than 44 percent of all school meals delivered daily. Several countries are now developing new universal programmes while others are considering switching to this approach; within 2024 researchers expect more than half of all programmes will be universal (2). Universal school meals programmes ensure distributional equity and improve children’s nutrition by steering them away from unregulated sources of food, whilst bringing down the overall cost per meal due to economies of scale. In addition, the universal delivery of meals supports social cohesion through shared meals and eliminates the stigma that arises from eligibility criteria. The list of countries currently delivering universal programmes includes those that have delivered universal programmes continuously for more than 70 years, including Finland, Sweden and Japan, and also the two largest programmes in the world: those of Brazil and India. Most recently, eight states in the USA have adopted universal school meals following their positive experience of delivering a universal programme in response to school closures during the COVID-19 pandemic, and the Community Eligibility Provision has had the effect of making universal school meals available in most cities across the nation. The key attractions of this approach are the long-term returns to human capital and social capital: Sweden found that students who had access to universal school meals had on average a 3 percent higher lifetime income than students who attended schools without this policy (3).

*The wellbeing of the learner is increasingly recognized as a key determinant of educational achievement.*

There is growing evidence that the wellbeing and education of school children both depend on a strong school platform which delivers a quality education and promotes health and wellbeing, resulting in a substantial increase in cross-sectoral policy guidance focused on school-age children and adolescence (4,5). The 2023 UNESCO report on school health, for example, emphasized the role of good health, nutrition, and wellbeing as essential to maximize education potential and provides the most comprehensive overview of school health and nutrition programmes and policies worldwide (6). Other agencies have similarly published guidance that bolster support for a holistic package of school health and nutrition interventions, including on the complementarities of health and education investments (e.g., Global Partnership for Education, WHO, etc.); the role of diets and food systems (e.g., FAO, UN Nutrition, WFP, etc.); and around the development of human capital (e.g., Global Financing Facility, USAID, World Bank Group, etc.) (7). Research on specific health concerns, such as malaria, has also shown that school-based interventions to prevent infection can improve cognitive skills and education outcomes by a similar order of magnitude to more direct education interventions (8) (Figure 4).


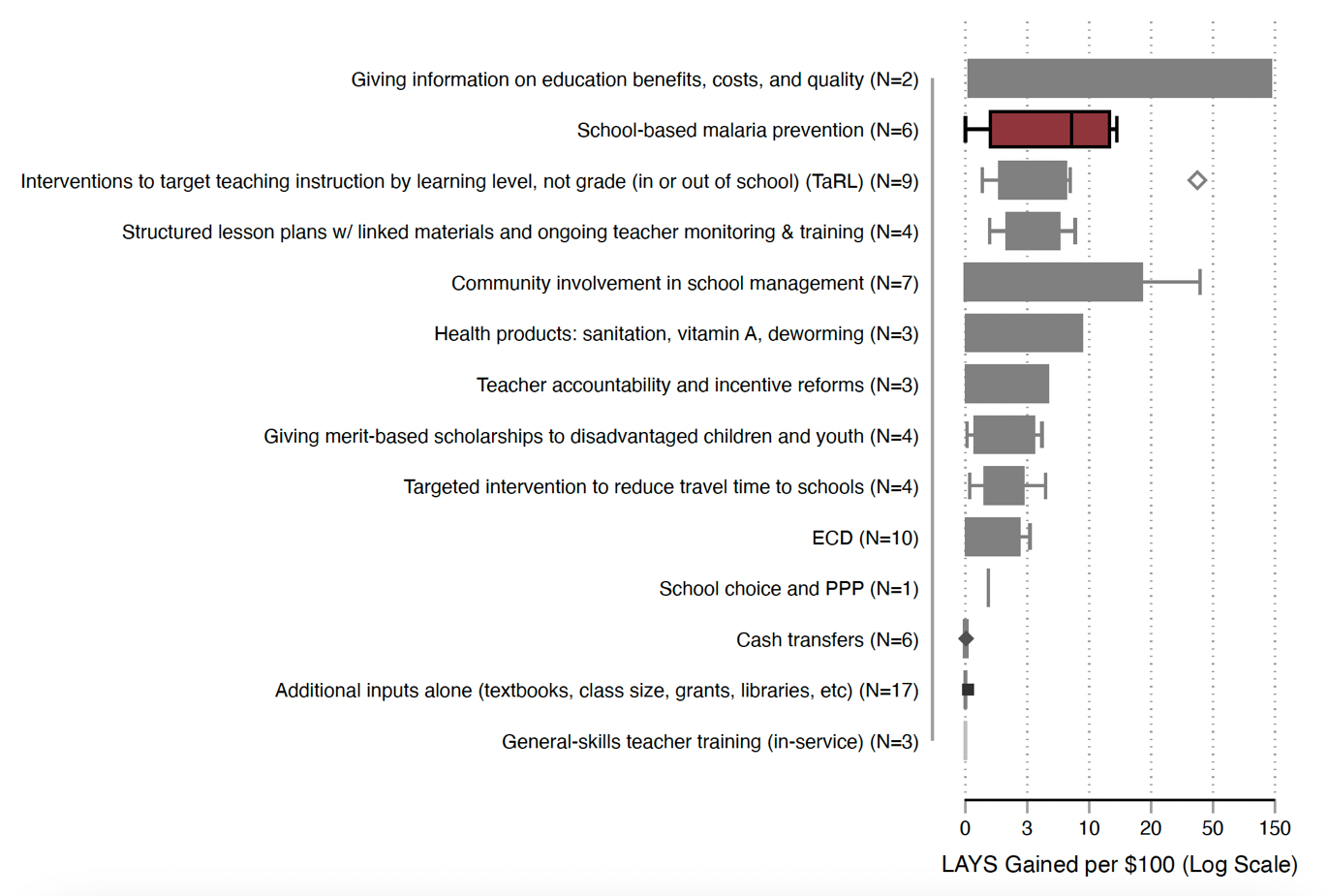
Figure 4. Cost-Effectiveness of School-Based Malaria Chemoprevention in Enhancing Cognitive Skills and Education Outcomes; Compared to the Cost-Effectiveness of Selected Education Interventions

Source: (8)

*New research on school-age children confirms the importance of good nutrition during the vulnerable phases of development throughout the “next 7,000 days”.*

Investing in the first 1,000 days of life is crucial for survival, and investments through the “next 7,000 days” are essential to secure wellbeing into adult life and for the next generation. Health and nutrition research to date has overwhelmingly focused on children under five years of age, representing some 40 percent to 80 percent of published health research on young people under 20 years. In contrast, only 10 percent of published research is about the health of school-age children (5 to 14 years of age), making the schoolchild the most neglected age-group in terms of research focus. This is important because neglect in research has resulted in the neglect and under-recognition of the health problems of this age group (7). Emerging research by nutritionists over the past 12 months has revealed the importance of nutrition interventions during the years when children are in school, and in particular, around puberty, to sustain wellbeing and healthy development. The importance of nutrition at school-age was specifically identified through the US NIH/USDA/Research Consortium BOND-KIDS project (9–13); the creation of special interest groups within the UK Nutrition Society to strengthen nutritional research in school-age children; and in Europe by the Hohenheim Declaration of the 2023 Hidden Hunger Conference (14). This has also shown the need to identify specific indicators for the nutrition of school-age children and adolescents, which aligns with the development of the Data and Monitoring Initiative led by WFP.

## New Insights into Effective Policies

*Policy changes to school meals programmes can immediately strengthen the community response to environment and climate and, through the power of procurement, help change agricultural practices in the longer term.*

A new White Paper produced by the Research Consortium shows that school meals offer this unique opportunity because of their reach and scale: in many countries school meals represent more than 70 percent of all publicly managed food systems. Two sets of policy changes are involved. The first group of policy changes are those directly controlled by national governments. Governments hold the policy levers of national programmes and can bring about changes that can have direct effects on critical areas that influence their young people now and throughout their lives. The biggest effects were found to be in the following four priority areas: menu changes which encourage dietary shifts that promote planetary and population health; clean and energy efficient cooking methods; prevention of food loss and waste, and reduction of plastic use; and action-oriented and holistic food education to help establish life-long healthier and more sustainable food practices. The second group of policy changes builds on the power of procurement to create demand for school foods from ecologically sustainable local farm systems, where possible, stimulating local approaches to agriculture which are regenerative, and which promote biodiversity, resilience, and food sovereignty. The policy influence here is less direct, and the power of procurement plays the major role if governments make the active and deliberate policy shifts in where they source school food. Planet-friendly dietary changes can be cost-neutral, having significant impacts on children’s nutrition and on carbon emissions at no extra cost. Some changes can directly reduce costs, such as moving to flexitarian diets from those based on some current food standards; switching from open fires to cooking stoves; and waste reduction that effectively reduces the per-capita cost of food (15).

*National school meals programmes successfully target multiple policy objectives, with health and nutrition, education and social protection among the most commonly reported aims.*

As shown in Figure 5, low-income countries typically target an average of three objectives, almost twice the scope of the 1.6 targeted by upper-middle-income countries. Health and nutrition is the most common objective in all income settings, and the relevance of non-health and nutrition objectives is inversely related to income level. In contrast, the policy relevance of education appears to decline as countries become wealthier. Income transfers are the third most common objective, and the relevance of transfers appears to decline with income level, perhaps because of a reduction in the vulnerable population decreases and the increased availability of other options for social assistance. This perspective broadens the value proposition of the role school meals programmes and expands the range of tools that countries have at their disposal to address several of the most common developmental challenges (16,17).

Figure 5. School meals objectives at a programme level, percentage of programmes (N=185)

| Objective | Low income | Lower-middle income | Upper-middle income | High income | Total |
| --- | --- | --- | --- | --- | --- |
| Education | 100.0 | 91.5 | 78.8 | 69.8 | 83.6 |
| Income transfer | 85.0 | 78.7 | 69.7 | 63.5 | 73.2 |
| Health and nutrition | 90.0 | 93.6 | 93.9 | 93.7 | 92.9 |
| Agriculture | 62.5 | 48.9 | 30.3 | 30.2 | 42.1 |
| Obesity | 5.0 | 17.0 | 30.3 | 68.3 | 34.4 |
| Average number of objectives per programme | 3.0 | 2.2 | 1.7 | 2.1 | 2.2 |

Source: (16,18)

*The most popular metric used by countries to assess their national school meals policies is the World Bank SABER policy tool.*

National school health and nutrition programmes experienced a decade of consistent growth up to 2020. To assist this process, the World Bank SABER (Systems Approach for Better Education Results) policy tool was introduced in 2012, and was included in the WFP policy for school meals in 2013. As shown in Figure 6, at least 81 low- and lower-middle-income countries have subsequently chosen this approach as their main policy tool to help design or strengthen their programmes. The tool is used to identify policy gaps and suggest solutions, and some countries have used the tool several times over sequential years to track and guide policy change. The results of the SABER reviews suggest that the tools were most relevant to strengthening programme design and implementation, as well as assessing the financial capacity of the programmes. The tool has now been in active use for 11 years, and the longevity of the SABER instrument together with its robust effectiveness in practical applications suggests that it continues to be the policy tool of choice by countries for strengthening the design of their national school meals programmes (19).

Figure 6. Cumulative number of SABER School Health and School Feeding exercises completed since 2012 globally and in sub-Saharan Africa, by year


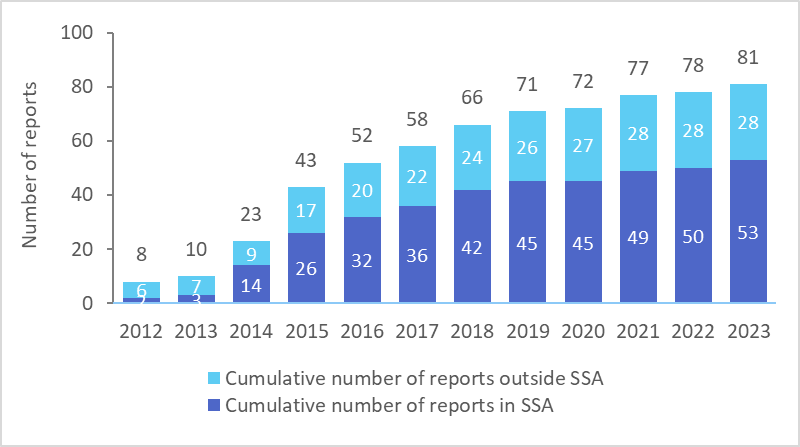


## Source: (19)

*Analysis of public policies in eight high-income countries identifies three common challenges and suggests three solutions that can help ensure equitable coverage.*

The Research Consortium’s “Good Examples” Community of Practice’s mandate is to conduct case studies of the national programmes in all member countries, with the longer term aim of using comparative analysis to identify common challenges and their solutions. To provide proof of principle, cases studies from an initial 9 countries have been reviewed (20–28). The review identified context-specific challenges to implementation fidelity related to three issues: transparency of national data, particularly around costs; the availability of food systems-based standards that consider sustainability; and accessibility for the most vulnerable students. The review suggests that solutions to these common challenges require: i) a consistent monitoring tool or methodology to track procurement, costs, and the impact of the school menus on sustainability targets; (ii) food systems-based school canteen standards to produce school meals with minimal waste, in line with sustainability objectives; and (iii) a of review eligibility criteria to increase school meals uptake among disadvantaged children. Currently 24 country case studies are underway or completed, and new studies are being commissioned.

## Translating New Insights into Policy

Based on the evidence available after two years of analysis, the Research Consortium for School Health and Nutrition calls upon the more than 95 member states of the School Meals Coalition to consider supporting the following priority areas of policy change moving forward:

1. **Institutionalize within existing national school meals programmes the policies identified in the White Paper “*School Meals and Food Systems:* *Rethinking the consequences for climate, environment, biodiversity and food sovereignty*”**, which seek to encourage nutritious and planet-friendly school meals sourced from ecologically sustainable agriculture. This includes policies that prioritize nutrient rich and plant-based school meals; efficient cooking methods in school kitchens; minimizing food waste and plastic pollution; and adopting a holistic food education curriculum. The paper also encourages a shift to school food procurement approaches that help promote regenerative agricultural practices that enhance biodiversity, agroecological approaches and food sovereignty.
2. **Prioritize health and nutrition research focused on school-age children** to improve the availability of evidence as well as monitoring and evaluation indicators to guide the design of school meals and complementary programmes that bolster human capital formation across the “next 7,000 days”.
3. **Agree new indicators to track the nutrition of school-age children and adolescents**, which can contribute to the ongoing discussion about the creation of an SDG indicator on school meals.
4. **Develop country case studies and value-for-money analyses across all School Meals Coalition member states** to document good examples from ministries, departments, and agencies in working across key sectors to deliver these multi-sectoral programmes efficiently and equitably. The case studies developed to date demonstrate that national case studies and value-for-money analyses are a useful learning tool for advancing our understanding of how to optimize school meals programme design, both for the country that is being studied, and for other Coalition member states.
5. **Consider whether the School Meals Coalition member states should adopt the World Bank SABER policy tool** as a way of tracking how current policies compare against good practice and as a tool to develop ambitious but realistic national commitments to improve and scale current national programming.

**References**

1. World Food Programme. State of School Feeding Worldwide 2022. Rome: World Food Programme; 2023.

2. Cohen JFW, Verguet S, Giyose BB, Bundy D. Universal free school meals: the future of school meal programmes? The Lancet. 2023 Aug;

3. Cohen JFW, Hecht AA, McLoughlin GM, Turner L, Schwartz MB. Universal School Meals and Associations with Student Participation, Attendance, Academic Performance, Diet Quality, Food Security, and Body Mass Index: A Systematic Review. Nutrients. 2021 Mar 11;13(3):911.

4. Bundy DAP, de Silva N, Horton S, Patton G, Schultz L, Jamison DT, et al. Investment in child and adolescent health and development: key messages from Disease Control Priorities, 3rd Edition. The Lancet. 2018;391(10121):687–99.

5. Kristjansson E, Osman M, Dignam M, Labelle PR, Magwood O, Galicia AH, et al. School feeding programs for improving the physical and psychological health of school children experiencing socioeconomic disadvantage. Cochrane Database of Systematic Reviews. 2022 Aug 24;2022(8).

6. UNESCO. Ready to learn and thrive: school health and nutrition around the world. Paris; 2023.

7. Schultz L, Hangoma P, Jamison DT, Bundy DAP. Cross-national experiences on child health and development during school-age and adolescence: the next 7,000 days. In: Norheim OF, Alwan A, editors. Disease Control Priorities. 4th ed. Washington, DC: World Bank; 2025.

8. Angrist N, Jukes MCH, Clarke S, Chico RM, Opondo C, Bundy DAP, et al. School-based malaria chemoprevention as a cost-effective approach to improve cognitive and educational outcomes: a meta-analysis. 2023 Mar 19;

9. Raiten DJ, Bundy DA, DeBernadro D, Steiber A, Papoutsakis C, Jimenez B, et al. Biomarkers of Nutrition for Development – Knowledge Indicating Dietary Sufficiency (BOND-KIDS) The BOND-KIDS Project: Executive Summary. Journal of Nutrition.

10. Black MM, Bruening Meg M, Carroll-Scott A, Dave JM, Ebi KL, Iannotti LL, et al. Biomarkers of Nutrition for Development (BOND)-KIDS: Understanding the nutrition ecology and emerging research gaps in nutrition that impact school-aged children and adolescents’ functioning. Journal of Nutrition.

11. Mehta S, Duggan CP, Addo Y, Zemel BS, Owino VO, Crouter SE, et al. Biomarkers of Nutrition for Development (BOND)-KIDS: Assessment. Journal of Nutrition.

12. Weaver CM, Georgieff MK, Giardi G, Kleinman R, Ramakrishnan U, Sacheck JM, et al. Biomarkers of Nutrition for Development (BOND)-KIDS: Understanding the biological evidence and emerging research gaps in nutrition that impact the health of school-aged children. Journal of Nutrition.

13. Economos C, Cohen J, Dary O, Debarnardo D, Giyose B, Herrera Cuenca M, et al. The Biomarkers of Nutrition for Development – Knowledge Indicating Dietary Sufficiency (BOND-KIDS): Examining effective Translation and Implementation methods for equitable access and scaling of nutrition programs. Journal of Nutrition.

14. The Hohenheim Declaration: Recommendations for School Meals Globally or “Plan for Action” [Internet]. Stuttgart; 2023. Available from: https://hiddenhunger.uni-hohenheim.de/en/hoh-decl

15. Pastorino S, Springmann M, Backlund U, Kaljonen M, Milani P, Bellanca R, et al. School meals and food systems: Rethinking the consequences for climate, environment, biodiversity and food sovereignty: A White Paper of the Research Consortium for School Health and Nutrition, an initiative of the School Meals Coalition. London; 2023.

16. Bedasso B. Center for Global Development. 2023. Not My Problem: Breaking Down Sectoral Silos in School Meals. Available from: https://www.cgdev.org/blog/not-my-problem-breaking-down-sectoral-silos-school-meals

17. Bundy DAP, Gentilini U, Schultz L, Bedasso B, Singh S, Okamura Y, et al. School Meals, Social Protection and Human Development: Revisiting Global Trends, Evidence, and Practices with a focus on South Asia. Washington, DC; 2024 Mar. (Social Protection & Jobs Discussion Paper Series). Report No.: 2401.

18. Global Child Nutrition Foundation (GCNF). School Meals Programs around the World: Results from the 2021 Global Survey of School Meal Programs [Internet]. 2022 [cited 2023 Jan 16]. Available from: https://survey.gcnf.org/2021-global-survey/

19. Schultz L, Renaud A, Bundy DAP, Barry FB, Benveniste L, Burbano C, et al. The SABER School Feeding Policy Tool: a Ten-Year Analysis of its Use by Countries in Developing Policies for their National School Meals Programs. Front Public Health. 2024;12(1337600):1–14.

20. Kuusipalo H, Manninen M. School Meals Case Study: Finland. London; 2023.

21. Bremner M, Defeyter G. School Meals Case Study: England. London; 2022.

22. Rueta AT, Tasala K, McKenna M, Martin A, Michnik K, Edwards G, et al. School Meals Case Study: Canada. London; 2023.

23. Toossi S, Cohen J, Clift J, Turner L, Gosliner W, Schwartz M. School Meals Case Study: United States of America. London; 2023.

24. Avallone S, Giner C, Nicklaus S, Darmon N. School Meals Case Study: France. London; 2023.

25. Brophy S, Woolley K. School Meals Case Study: Wales. London; 2022.

26. Furey S, Woodside J. School Meals Case Study: Northern Ireland. London; 2022.

27. Ministry of Agriculture F and F, Ministry of Education CSS and T. School Meals Case Study: Japan. London; 2023.

28. Brennan M, Jones J, McKendrick JH. School Meals Case Study: Scotland. London; 2022.
